# First Total-Body Kinetics Study of ^18^F-Flurpiridaz in Healthy Volunteers at Rest and Feasibility of Simplified Quantitative Strategies

**DOI:** 10.2967/jnumed.125.270997

**Published:** 2026-07

**Authors:** Hao Song, Zhenyu Xiang, Yihan Wang, Jingxu Zhao, Yongpeng Wen, Zhixing Qin, Jia Gao, Zhifang Wu, Ping Wu, Sijin Li

**Affiliations:** 1 Department of Nuclear Medicine, First Hospital of Shanxi Medical University, Taiyuan, China;; 2 Collaborative Innovation Center for Molecular Imaging of Precision Medicine, Shanxi Medical University, Taiyuan, China;; 3 Treatment & Transformation of Nuclear Medicine, CAEA Center of Excellence on Nuclear Technology Applications for Diagnosis, Taiyuan, China;; 4 United Imaging Healthcare Co., Ltd., Shanghai, China; and; 5 ShanghaiTech University, Shanghai, China

**Keywords:** ^18^F-flurpiridaz, total-body PET, pharmacokinetics, simplified quantification, mitochondrial complex I

## Abstract

The multiorgan kinetic profile of ^18^F-flurpiridaz targeting mitochondrial complex I remains inadequately characterized. We aim to characterize preliminary total-body pharmacokinetics of ^18^F-flurpiridaz in healthy volunteers and evaluate shortened acquisition protocols for clinical translation. **Methods:** Twelve healthy volunteers were imaged with ^18^F-flurpiridaz during a 60-min dynamic total-body PET/CT scan on the uEXPLORER scanner at rest. Time–activity curves were derived from volumes of interest generated by an automated CT-based segmentation method applied to motion-corrected dynamic PET images, with manual delineation of the descending aorta, kidney, and breasts. The descending aorta served as the input function for most organs, whereas the pulmonary artery was used for the lungs. The 2-tissue irreversible (2T3K) and 2-tissue reversible (2T4K) compartment models incorporating blood volume and time-delay correction were compared using the Akaike information criterion (AIC). Simplified metrics of distribution volume (*V*_T_) from a Logan plot and SUV_mean_ from truncated scans (10, 30, and 60 min) were correlated with the reference 60-min 2T4K *V*_T_. **Results:** Four distinct kinetic patterns were observed across different organs. At 60 min, the 2T4K model demonstrated superior fitting performance (lower AIC) in 15 of 18 targeted regions. The 2T3K model exhibited lower AIC in the heart, brain, and kidneys at 10 min. In the thyroid and spinal cord, the optimal model shifted from the 2T3K model at 60 min to the 2T4K model at 10 min. Logan *V*_T_ from 30-min (*r* = 0.974) and 60-min (*r* = 0.979) scans strongly correlated with reference *V*_T_. Notably, SUV_mean_ around 10-min postinjection also showed strong correlation with reference *V*_T_ (*r* = 0.834–0.909), only slightly lower than that observed in the optimal 25–30-min window (*r* = 0.922; all *P* < 0.0001). **Conclusion:** This total-body kinetic atlas of ^18^F-flurpiridaz supports the use of the 2T4K model for multiorgan quantification, time-dependent model preference, and the feasibility of ultrashort imaging strategies for clinical mitochondrial complex I assessment, establishing a foundation for future applications in oncology (e.g., metabolism evaluation), neurology (e.g., mitochondrial mapping), and integrated cardio-oncology diagnostics.

## Visual Abstract

**Figure:**
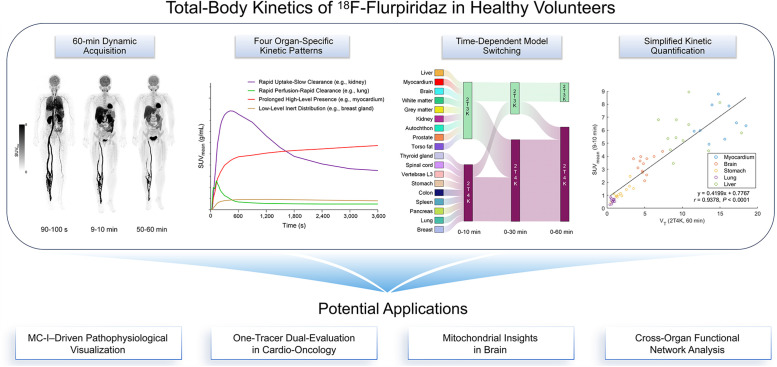

Initially developed for myocardial perfusion imaging, ^18^F-flurpiridaz is a novel positron-emitting radiotracer offering high imaging quality and a favorable pharmacokinetic profile (1–3) without affecting myocardial cell viability (4). Its molecular target, mitochondrial complex I (MC-I), is the initial enzyme complex of the mitochondrial electron transport chain and is implicated in tumor metabolic reprogramming (5) and aggressive progression (6). This supports MC-I as a critical regulatory node and a promising metabolic biomarker in oncology, particularly for tumors with predominant oxidative metabolism, low glycolytic activity, and therapy-resistant metabolic pathways (7,8). A recent study successfully used dual-time-point ^18^F-flurpiridaz PET/CT to localize parathyroid adenomas in patients with primary hyperparathyroidism (9), providing preliminary evidence for its utility in tumor imaging and other pathologic conditions. Beyond oncology, MC-I is also fundamental to mitochondrial structural dynamics in the brain (10), affecting neural networks and leading to cognition and behavior alteration (11) and providing valuable insights into neurologic disorders, such as neuroinflammation (12).

Absolute quantification of ^18^F-flurpiridaz kinetics across the entire body is critical to unlocking its potential for multiorgan and cross-disease applications. Although previous studies demonstrated various protocols to accurately estimate absolute myocardial blood flow derived from regional dynamic ^18^F-flurpiridaz imaging (13–15), the limited axial coverage of conventional PET/CT scanners (16) hindered a comprehensive evaluation of total-body ^18^F-flurpiridaz kinetics. The advent of long–axial-field-of-view PET/CT systems provides a feasible platform for integrated multifacet kinetic profiles of molecular imaging (17–19), which may lead to a better understanding of both perfusion and MC-I distribution based on ^18^F-flurpiridaz across the human body.

Herein, to the best of our knowledge, we present the first single-session, total-body dynamic ^18^F-flurpiridaz PET/CT study of healthy volunteers at rest. We aim to systematically characterize time–activity curves across organs, determine the most appropriate pharmacokinetic models for different scan duration, establish an organ-specific kinetic parameter atlas, and develop simplified quantitative strategies to facilitate cross-disease translational applications of ^18^F-flurpiridaz.

## MATERIALS AND METHODS

### Study Participants

In total, 14 healthy volunteers with no history of malignancy, cardiovascular or cerebrovascular disease, or active inflammation were enrolled after comprehensive medical history review and physical examination. Each subject underwent a 60-min dynamic ^18^F-flurpiridaz total-body PET/CT scan. Two participants were excluded: one male because of scan intolerance at 40 min and one female with incidental pulmonary adenocarcinoma. The remaining 12 participants were included in the final analysis. Given the gradual increase in myocardial uptake observed during later acquisition phases, we obtained supplementary prolonged (4 h) acquisition data from one newly recruited volunteer.

This prospective study was approved by the institutional review board of the First Hospital of Shanxi Medical University (approval no. KYLL-2025-161) and was conducted in accordance with the principles of the Declaration of Helsinki. Written informed consent was obtained from all participants before enrollment.

### Radiopharmaceutical Preparation

The radiotracer was synthesized using a CFN-MPS-100 fluorine multifunction radiopharmaceutical synthesis module (Sumitomo Heavy Industries), mainly following the procedures described in previous studies (4,20,21). Detailed synthesis procedures are provided in the supplemental materials (available at http://jnm.snmjournals.org).

### Dynamic Total-Body PET/CT Acquisition and Reconstruction

**FIGURE 1.**
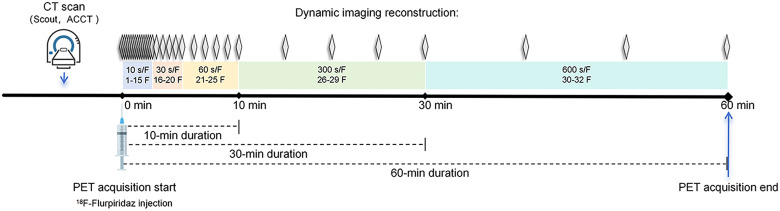
Total-body dynamic ^18^F-flurpiridaz PET/CT acquisition and reconstruction protocol.

All participants underwent dynamic total-body PET/CT on the uEXPLORER (United Imaging Healthcare) scanner (Fig. 1). Dynamic PET data were binned into 32 frames (15): 15 × 10 s/frame, 5 × 30 s/frame, 5 × 60 s/frame, 4 × 300 s/frame, and 3 × 600 s/frame. Detailed participant preparation, acquisition protocol, and image reconstruction parameters are provided in the supplemental materials (22).

### Dynamic PET Image Processing

Total-body motion occurring after 5 min in all participants was first corrected using the Fast Algorithm for Motion Correction (23). The detailed organ segmentation process for the volumes of interest analysis is provided in the supplemental materials (24).

Considering the typical 0–10-min acquisition window used for myocardial flow quantification and the commonly used 30-min duration in cardiac PET/MR protocols, we selected 2 additional time windows to explore the feasibility of short-time acquisition quantitative strategies. Time–activity curves were then extracted for all volumes of interest from 10-, 30-, and 60-min dynamic images.

### Kinetic Modeling Fitting and Correlation Analysis for Simplified Quantitative Strategies

All time–activity curve data were imported into the PKIN tool of PMOD version 4.4 (PMOD Technologies) for model fitting using an irreversible 2-tissue compartmental model (2T3K) with rate constants *K*_1_, *k*_2_, and *k*_3_ and a reversible 2-tissue compartmental model (2T4K) with rate constants *K*_1_, *k*_2_, *k*_3_, and *k*_4_. Tissue blood volume fractions and time-delay correction were applied in all model fittings. The Akaike information criterion (AIC) was used to determine the most appropriate compartmental model, whereas *R*^2^ was used to evaluate the goodness of fit of different organs with each kinetic model. With the optimal model, the net influx rate (*K_i_*) from 2T3K or the total distribution volume (*V*_T_) from 2T4K was obtained.

The pulmonary artery was used as the input function for analysis of lung kinetics because of lower AIC compared with the descending aorta (18.85 ± 40.71 vs. 80.68 ± 59.74; Supplemental Table 1). The descending aorta was used as the input function for the other organs. Time-delay correction was applied in all model fittings. Dual-blood kinetic modeling of the liver implemented in MATLAB (2023a; The MathWorks) is provided in the supplemental materials (25).

For simplified quantification strategies, the graphical analysis using the Logan method was performed at a time of 10 min because of its higher goodness of fit (*R*^2^ = 0.997 ± 0.004) in most organs compared with Patlak analysis (*R*^2^ = 0.660 ± 0.221; Supplemental Table 2). Additionally, the correlation between Logan *V*_T_ and the SUV_mean_ at multiple time points with the reference 60-min 2T4K *V*_T_ was analyzed.

### Statistical Analysis

All statistical analyses were performed using SPSS Statistics version 26.0 (IBM). Data are presented as mean ± SD. The variability of the tracer distribution within the cohort is described by the coefficient of variation, calculated as the ratio of SD and the mean, multiplied by 100%. Correlation was assessed using the Spearman rank correlation coefficient. To identify phases of relatively stable myocardial uptake, uptake differences across 3 intervals (within 30–60 min) were compared using a repeated-measures ANOVA. *P* values of less than 0.05 were considered statistically significant.

## RESULTS

Among the 12 healthy volunteers, there were 6 men and 6 women, with a mean age of 47.1 y and a mean body mass index of 23.7 kg/m^2^. Demographic characteristics are summarized in Table 1.

**TABLE 1.** Basic Information of 12 Healthy Participants

Subject	Sex	Age (y)	Height (cm)	Weight (kg)	BMI (kg/m²)	Injected dose (MBq)
1	Female	25	168	60	21.3	132.0
2	Male	25	176	70	22.6	133.9
3	Male	32	185	83	24.3	120.3
4	Female	35	171	65	22.2	116.0
5	Male	38	172	82	27.7	138.4
6	Male	39	174	72.5	23.9	119.4
7	Female	52	168	67.5	23.9	120.3
8	Male	57	167	63	22.6	131.2
9	Female	60	160	55	21.5	119.9
10	Female	61	165	70	25.7	113.8
11	Male	70	171	70	23.9	149.8
12	Female	71	156	60	24.7	116.3
Total average		47.1 ± 16.7	169.4 ± 7.5	68.2 ± 8.4	23.7 ± 1.8	125.9 ± 11.0

BMI = body mass index.

### Organ-Specific Kinetic Patterns of ^18^F-Flurpiridaz

**FIGURE 2.**
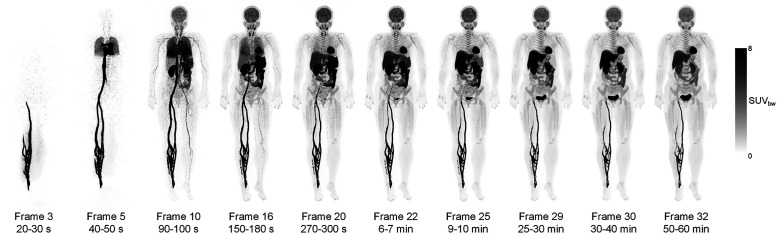
Maximum-intensity projection of dynamic PET images of a representative female volunteer.

**FIGURE 3.**
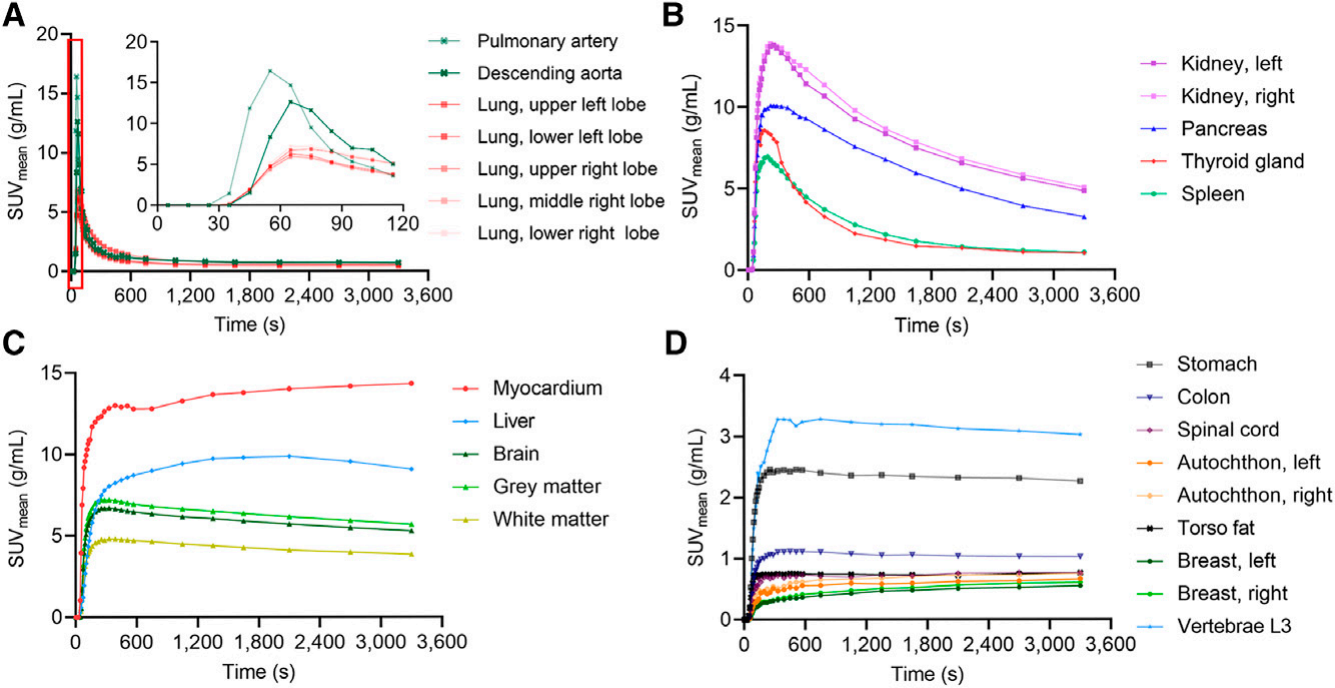
Time–SUV curves of ^18^F-flurpiridaz from a representative female participant.

Dynamic PET images of one representative female volunteer are demonstrated in Figure 2. Four dynamic radiotracer uptake patterns were identified on the basis of visual evaluation of time–activity curve kinetics (uptake rate, peak formation, clearance trend). (1) Rapid perfusion–rapid clearance (Fig. 3A, for example, lungs): fast blood-driven uptake peaks appeared within seconds to 1 min, followed by rapid clearance via blood redistribution, showing a “steep rise–sharp decline” curve. (2) Rapid uptake–slow clearance (Fig. 3B, for example, pancreas, kidneys, thyroid gland, spleen): an early peak appeared (1–4 min) with gradual clearance due to slow metabolism and excretion, producing a “fast peak–broad decline” curve. (3) Prolonged high-level presence (Fig. 3C): this pattern was characterized by a fast and high-level distribution within minutes, followed by a phase of very gradual accumulation (e.g., myocardium) or slow decline (e.g., liver and brain), resulting in overall prolonged retention. Myocardial uptake showed no statistically significant difference between the 40–50- and 50–60-min frames, peaked at 60 min, and subsequently declined gradually (Supplemental Fig. 1). (4) Low-level inert distribution (Fig. 3D, for example, stomach, colon, spinal cord, vertebra, torso fat, breasts): this pattern showed persistently low, flat radioactivity (low tracer affinity/metabolic rate), reflecting “inert distribution” with minimal fluctuation.

Individual organ time–activity curves derived from SUV_mean_ are shown in Supplemental Figures 2–5. The coefficient of variation of the time–activity curves differed among organs such as the myocardium, brain, and vertebra and those such as the stomach and thyroid. Moreover, within the same organ, it varied from early to late postinjection phases. At 5 min postinjection, tissues including heart, brain, fat, and spinal cord exhibited the lowest uptake variability (Supplemental Fig. 6).

### Kinetic Rate Constants of 2T4K Modeling Derived from 60-Minute Scan Duration

For the 60-min full scan duration, the 2T4K model demonstrated lower AIC values in 15 of 18 tissues compared with the 2T3K model (18.99 ± 33.23 vs. 30.43 ± 23.44; Supplemental Table 3). Typical kinetic fitting curves are shown in Supplemental Figure 7. Descriptive statistics for all full-time kinetic rate constants are summarized in Table 2. All fitting models demonstrated excellent goodness of fit. Substantial interorgan variability was observed in kinetic parameters. The myocardium exhibited a high *K*_1_/*k*_2_ ratio (∼2.10), indicating predominant influx over efflux. In contrast, lung tissue showed a ratio of less than 1, consistent with rapid clearance. The *k*_3_/*k*_4_ ratio, reflecting intracellular binding stability, was highest in the spleen (∼2.63), moderate in the kidneys (∼1.22–1.31), and near 1 in white matter and adipose tissue, suggesting primarily nonspecific binding in the latter. High *V*_T_ was observed in kidneys (16.962 mL·cm^−3^), myocardium (16.659 mL·cm^−3^), pancreas (7.665 mL·cm^−3^), and brain (5.003 mL·cm^−3^), indicating strong uptake and affinity for MC-I. Conversely, tissues such as adipose, breasts, and upper lung lobes had a *V*_T_ of less than or equal to 1 mL·cm^−3^, reflecting limited tracer retention.

**TABLE 2.** Kinetic Constants Based on 2T4K Modeling of 12 Healthy Participants

Organs	*v*_B_ (%)	*K*_1_ (mL/cm^3^/min)	*k*_2_ (min^−1^)	*k*_3_ (min^−1^)	*k*_4_ (min^−1^)	*V*_T_ (mL/g)	*R* ^2^
Brain	4.20 ± 1.69	0.350 ± 0.044	0.215 ± 0.060	0.445 ± 0.289	0.105 ± 0.068	5.003 ± 1.009	0.991 ± 0.010
White matter	2.78 ± 1.33	0.224 ± 0.036	0.203 ± 0.092	0.265 ± 0.165	0.136 ± 0.056	3.427 ± 0.601	0.991 ± 0.011
Gray matter	4.89 ± 1.57	0.384 ± 0.051	0.202 ± 0.069	0.223 ± 0.133	0.141 ± 0.049	5.272 ± 1.426	0.992 ± 0.011
Thyroid gland	16.84 ± 17.92	1.408 ± 0.790	0.474 ± 0.175	0.615 ± 0.596	0.558 ± 0.269	3.118 ± 1.005	0.937 ± 0.045
Lung							
LUL	18.69 ± 9.63	1.367 ± 0.583	1.758 ± 0.523	0.545 ± 0.697	1.382 ± 0.799	0.752 ± 0.199	0.987 ± 0.015
LLL	23.48 ± 8.53	1.508 ± 0.423	1.535 ± 0.409	0.449 ± 0.526	1.134 ± 0.829	1.292 ± 0.363	0.993 ± 0.008
RUL	19.89 ± 10.54	1.318 ± 0.695	1.687 ± 0.382	0.594 ± 0.903	1.486 ± 0.918	0.757 ± 0.221	0.989 ± 0.015
RML	17.92 ± 4.28	1.190 ± 0.562	1.595 ± 0.171	0.580 ± 0.582	1.604 ± 0.891	0.739 ± 0.209	0.994 ± 0.007
RLL	24.09 ± 6.82	1.439 ± 0.430	1.480 ± 0.397	0.382 ± 0.451	0.694 ± 0.474	1.343 ± 0.423	0.996 ± 0.007
Myocardium	18.75 ± 7.31	0.668 ± 0.155	0.132 ± 0.064	0.180 ± 0.193	0.080 ± 0.040	16.659 ± 3.805	0.981 ± 0.020
Breast							
Left	0.06 ± 0.14	0.023 ± 0.005	0.213 ± 0.080	0.468 ± 0.221	0.053 ± 0.011	1.246 ± 0.680	0.998 ± 0.002
Right	0.03 ± 0.06	0.021 ± 0.005	0.268 ± 0.243	0.456 ± 0.199	0.048 ± 0.014	1.270 ± 0.618	0.995 ± 0.004
Liver	0.13 ± 0.26	0.375 ± 0.095	0.075 ± 0.091	0.151 ± 0.457	1.227 ± 1.263	10.726 ± 3.205	0.949 ± 0.028
Stomach	0.57 ± 0.79	0.138 ± 0.060	0.352 ± 0.183	0.389 ± 0.286	0.111 ± 0.044	1.908 ± 0.946	0.983 ± 0.014
Spleen	9.59 ± 10.11	1.327 ± 0.358	0.457 ± 0.206	0.710 ± 0.832	1.288 ± 1.044	3.501 ± 0.563	0.984 ± 0.014
Pancreas	7.19 ± 8.67	1.091 ± 0.249	0.475 ± 0.185	1.074 ± 0.881	0.424 ± 0.228	7.665 ± 1.876	0.984 ± 0.012
Kidney							
Left	15.08 ± 8.15	2.463 ± 0.346	0.401 ± 0.273	0.661 ± 0.742	0.525 ± 0.523	16.151 ± 3.753	0.981 ± 0.014
Right	16.71 ± 11.71	2.568 ± 0.643	0.308 ± 0.132	0.506 ± 0.512	0.728 ± 0.549	16.962 ± 6.221	0.977 ± 0.017
Prostate	1.06 ± 1.82	0.141 ± 0.087	0.127 ± 0.141	0.316 ± 0.357	0.222 ± 0.224	1.722 ± 0.631	0.984 ± 0.013
Colon	0.63 ± 0.83	0.103 ± 0.025	0.209 ± 0.064	0.480 ± 0.262	0.058 ± 0.017	1.921 ± 1.256	0.983 ± 0.024
Spinal cord	0.73 ± 0.89	0.057 ± 0.013	0.201 ± 0.113	0.240 ± 0.183	0.082 ± 0.040	1.131 ± 0.142	0.975 ± 0.021
Autochthon							
Left	0.20 ± 0.39	0.047 ± 0.019	0.356 ± 0.450	0.438 ± 0.321	0.132 ± 0.139	1.426 ± 0.090	0.967 ± 0.039
Right	0.40 ± 0.39	0.043 ± 0.016	0.319 ± 0.303	0.430 ± 0.474	0.159 ± 0.222	1.289 ± 0.353	0.974 ± 0.026
Torso fat	0.60 ± 0.67	0.092 ± 0.026	0.429 ± 0.272	0.400 ± 0.230	0.078 ± 0.031	1.371 ± 0.265	0.975 ± 0.022
Vertebrae	0.36 ± 0.55	0.139 ± 0.031	0.088 ± 0.052	0.321 ± 0.357	0.669 ± 0.770	3.152 ± 0.834	0.992 ± 0.009

LUL = left upper lung; LLL = left lower lung; RUL = right upper lung; RML = right middle lung; RLL = right lower lung; *v*_B_ = blood volume fraction.

### Model Transition at Different Time Intervals

**FIGURE 4.**
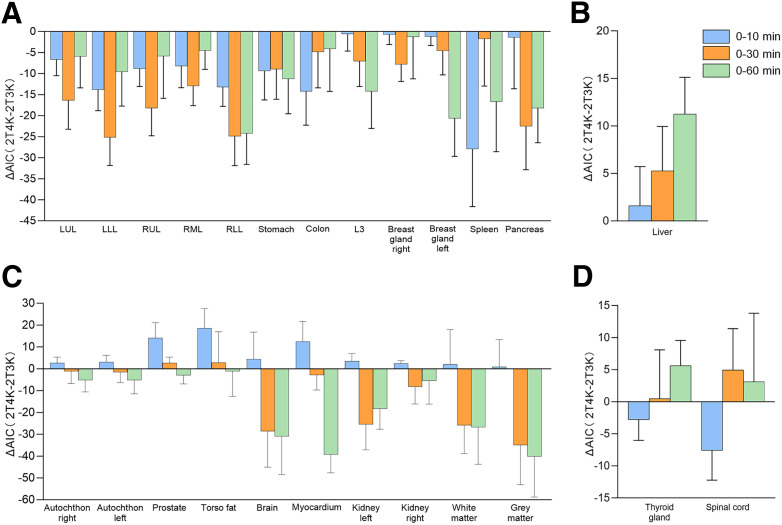
Differences in AIC values between 2T4K and 2T3K models across 10-, 30-, and 60-min acquisition durations. Center lines in box plots represent mean differences, and error bars indicate standard errors of differences. L3 = vertebrae L3; LLL = left lower lung; LUL = left upper lung; RLL = right lower lung; RML = right middle lung; RUL = right upper lung.

Comparative analysis of AIC values across time intervals revealed temporal heterogeneity in optimal model selection across organs, mainly manifested as 4 types: (1) sustained preference for the 2T4K model, for example, the spleen, pancreas, vertebra, gastrointestinal tract, breasts, and lungs (Fig. 4A); (2) sustained preference for the 2T3K model, for example, the liver (Fig. 4B; model transition results of the dual-blood-supply liver model are presented in Supplemental Tables 4 and 5 and Supplemental Figs. 8 and 9); (3) transition from 2T3K (early) to 2T4K (late) dominance, for example, autochthonous muscle, prostate, torso fat, brain, myocardium, and kidney (Fig. 4C); (4) transition from 2T4K to 2T3K dominance, for example, the thyroid gland and spinal cord (Fig. 4D).

### Feasibility of Short Time Scan in Quantifying MC-I Distribution

For linear graphical analysis, Logan *V*_T_ from the 30- and 60-min datasets exhibited similarly strong correlations with the reference *V*_T_ (*r* = 0.979 and 0.974, respectively; *P* < 0.0001). When the acquisition time shortened to 10 min, the correlation declined moderately but still remained significant (*r* = 0.826, *P* < 0.0001).

**FIGURE 5.**
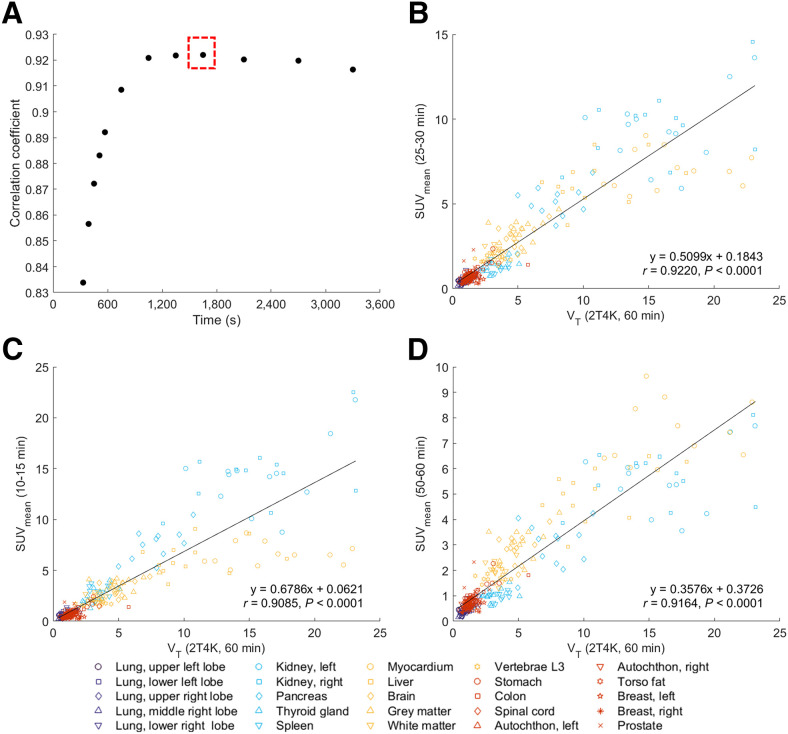
Correlation between SUV_mean_ and reference *V*_T_ (2T4K, 60 min) across time windows.

The correlation of SUV_mean_ with the reference *V*_T_ is shown in Figure 5A. The correlation coefficient peaked at 25–30 min (*r* = 0.922; Fig. 5B) and remained robust at 10–15 min (*r* = 0.909; Fig. 5C) and 50–60 min (*r* = 0.916; Fig. 5D) (all *P* < 0.0001). The correlations across the remaining time windows are illustrated in Supplemental Figure 10, exhibiting high correlation coefficients between 0.834 and 0.922.

## DISCUSSION

This study represents the first comprehensive evaluation of total-body ^18^F-flurpiridaz imaging for resting-state pharmacokinetic modeling in healthy volunteers. It uniquely features the feasibility of accurately quantifying MC-I with shortened acquisition durations, laying the foundation for clinical translation of ^18^F-flurpiridaz from cardiac-specific to multiorgan applications. This work fosters a paradigm shift from traditional organ-specific (“point”) assessments to a more integrated, system-level (“plane”) perspective in molecular imaging.

Previous ^18^F-flurpiridaz biodistribution studies using effective dose measurements (20,26) and semiquantitative SUV parameters (27) consistently reported high uptake in the myocardium, brain, liver, and kidneys. This aligns with our findings, in which these organs exhibited substantially higher *V*_T_ values than others. Our study also revealed profound temporal heterogeneity in kinetic patterns and model selection across organs. The sustained tracer accumulation in the myocardium, brain, and kidneys, from the 2T3K model (10 min) to 2T4K model (60 min), aligns with high metabolic demand and sustained MC-I binding, underscoring that even in organs with high mitochondrial density, early kinetics can be perfusion-influenced. In contrast, organs such as the lungs, stomach, and spleen were consistently fitted by the 2T4K model, indicating ongoing reversible binding dynamics. Interestingly, the thyroid and spinal cord demonstrated a reverse pattern, which may reflect rapid early-phase binding that reaches a transient equilibrium. The liver remained uniquely described by the 2T3K model throughout, aligning with its role in metabolism and biliary excretion. Therefore, future clinical applications should account for temporal heterogeneity in organ-specific kinetic models, and further investigation is needed to determine whether these patterns remain applicable under pathologic conditions.

Our results regarding a myocardium modeling shift supports previous studies that applied the irreversible 2T3K model for myocardial blood flow quantification with 20–30-min dynamic ^18^F-flurpiridaz imaging protocols (13,14) and validates the use of the reversible 2T4K model in murine studies using scan durations of 0–40 and 40–80 min (28). Note that 1-tissue compartment models were not assessed in this study, as prior murine research has demonstrated that such models fail to provide an adequate fit for myocardial ^18^F-flurpiridaz kinetics (28). Therefore, the observed transition to the 2T4K model in myocardium suggests that prolonged imaging could uniquely assess mitochondrial functional integrity via the reversibility parameter *k*_4_ beyond perfusion. It is worth noting that, in ischemic territories, alterations in *k*_4_ might thus be key to differentiating viable from infarcted tissue. Furthermore, the slowly rising myocardial activity observed within 60 min postinjection not only necessitates a reevaluation of the 1-d stress/rest protocol—particularly ensuring a sufficient injection interval (>40 min) to avoid underestimation of rest-phase residual activity—but also warrants caution against its potential misinterpretation as filling or redistribution, raising critical questions for future research: the timing of divergence in time–activity curves between normal and diseased myocardium and the clinical significance of such redistribution kinetics.

Our findings support the feasibility of reduced scan duration to enable simplified quantification in future clinical practice. The 25–30-min window may represent an optimal pseudoequilibrium phase for ^18^F-flurpiridaz distribution across diverse tissues. During this window, tissue uptake and clearance appear transiently balanced, allowing SUV to serve as a reliable surrogate for full kinetic quantification. In contrast, the 50–60-min window exhibited reduced agreement with *V*_T_, potentially due to increased tracer washout, declining plasma activity, and metabolite accumulation, resulting in deviation from equilibrium. Importantly, the sub–10-min window, in which all coefficients remained above 0.83, indicates sufficient robustness for simplified clinical quantification.

MC-I has been shown to play a pivotal regulatory role in various redox-dominant tumors (29,30). Therefore, the pharmacokinetic profile of ^18^F-flurpiridaz established in this study offers the potential for dual-purpose imaging in one single scan, simultaneously assessing myocardial perfusion and characterizing tumor metabolic pathways across different organs or tissues, thus providing a novel one-stop diagnostic paradigm targeting cardio-oncology comorbidities and cross-organ networks in future clinical practice. Crucially, in the one excluded participant with an incidental pulmonary adenocarcinoma, the ^18^F-flurpiridaz SUV_max_ measured at 5–10 min postinjection was 2.33, with an SUV_mean_ of 0.36 in normal lung tissue (Supplemental Fig. 11). This observation provides preliminary but compelling evidence supporting the robustness of the proposed paradigm.

On the other hand, our results may provide mitochondrial profiles across the human body. Previous in vitro and in vivo assays confirmed the lipophilicity (31) of ^18^F-BMS (logD7.4 = 3.69) within the appropriate range of logD7.4 for blood–brain barrier penetration and the specific binding of ^18^F-flurpiridaz to MC-I against inhibition with rotenone in the brain (32). Therefore, *V*_T_ was chosen to represent binding to MC-I in the brain from an ^18^F-BCPP-EF rat study (32). Notably, a recent study of brain mitochondrial profile using a physical voxelization approach to partition a frozen human coronal hemisphere section demonstrated that, compared with white matter, gray matter contains more than 50% more mitochondria (11). Our study revealed a similar gray matter–to–white matter 2T4K *V*_T_ ratio of approximately 1.54:1. The total-body ^18^F-flurpiridaz PET/CT imaging enables the construction of a quantitative mitochondrial distribution atlas across human organs, providing a valuable foundation for investigating the pathophysiology and progression of mitochondrial-related diseases.

The spatiotemporal heterogeneity of the coefficient of variation of SUV_mean_ time–activity curves originates from multiple sources: intersubject physiologic variability (e.g., cardiac output), organ-specific physiology (e.g., gastric motility), pharmacokinetic properties of ^18^F-flurpiridaz (e.g., flow-dependent first-pass extraction), and technical factors (e.g., partial-volume effects, motion, injection-scan delay). To enhance the robustness of multiorgan assessment, future quantitative protocols (e.g., tailoring acquisition timing or using correction methods) should account for this heterogeneity to improve accuracy and interpretability.

This study has several limitations. (1) Organ-specific modeling: while more sophisticated models accounting for tissue-specific factors could improve quantitative accuracy in some regions (33,34), as suggested by our preliminary analyses of a dual-blood-supply liver model, the arterial-only input remains reasonable because of hepatic artery dominance in lesion perfusion. For the lungs, the observed activity may be attenuated by air spaces (35), which was not corrected in this study. (2) Motion correction: we implemented an image-based dynamic PET motion-correction algorithm to address the motion artifacts, but we failed to resolve the attenuation errors due to PET and CT mismatch (22). Future studies should implement more rigorous motion correction to enhance quantitative accuracy, especially for respiratory-affected organs. (3) Metabolite correction: rising myocardial uptake and late-phase overestimation in high-perfusion organs suggest the presence of metabolites with distinct kinetic profiles and tissue affinity, warranting future integrated metabolite analysis to improve accuracy. (4) Partial-volume effects: no partial volume correction was applied, potentially affecting smaller volumes of interest. Large-matrix reconstruction (256 × 256 matrix) likely mitigated the effect to some extent. (5) Intergroup variation: Sex- and age-based subgroup analyses were not performed. Larger future cohorts are needed to validate the robustness of the proposed simplified quantitative method across diverse populations, particularly to assess demographic variability and its performance in specific patient groups such as those with oncologic or cardiovascular conditions.

## CONCLUSION

This study presents the first systematic evaluation of the kinetic characteristics of ^18^F-flurpiridaz across multiple organs using total-body dynamic PET imaging, extending the application of this mitochondrial functional tracer beyond myocardial imaging. Additionally, by comparing the suitability of different acquisition durations and kinetic models across various tissues, this work provides theoretic support for optimizing imaging protocols and facilitating clinical translation. These findings establish a technical foundation for multiorgan quantitative analysis and integrated cardio-oncology molecular imaging.

## DISCLOSURE

This study was supported by the National Natural Science Foundation of China (U22A6008), Fundamental Research Program of Shanxi Province (202503021211276), and Shanxi Province Higher Education “Billion Project” Science and Technology Guidance Project (BYYX001). No other potential conflict of interest relevant to this article was reported.

## Notes

KEY POINTS **QUESTION:** What are the pharmacokinetic characteristics of ^18^F-flurpiridaz across organs in healthy volunteers? Is the simplified quantification strategy for MC-I feasible by shortening the acquisition time? **PERTINENT FINDINGS:** The 2T4K model is the most appropriate model for evaluating the pharmacokinetics of ^18^F-flurpiridaz in the majority of human organs. With decreasing acquisition duration, several organs exhibited a time-dependent transition in model applicability. Using 2T4K *V*_T_ derived from 60-min data as the reference, strong correlations were observed for both Logan *V*_T_ and SUV_mean_ obtained from various acquisition windows across multiple organs. **IMPLICATIONS FOR PATIENT CARE:** This approach holds promise for advancing personalized patient care through MC-I–targeted visualization of disease processes, integrated cardio-oncology assessment, brain mitochondrial profiling, and cross-organ network-level diagnostics.
